# Inhibitory Potential of Five Traditionally Used Native Antidiabetic Medicinal Plants on ****α****-Amylase, ****α****-Glucosidase, Glucose Entrapment, and Amylolysis Kinetics *In Vitro*

**DOI:** 10.1155/2014/739834

**Published:** 2014-03-02

**Authors:** Carene M. N. Picot, A. Hussein Subratty, M. Fawzi Mahomoodally

**Affiliations:** Department of Health Sciences, Faculty of Science, University of Mauritius, 230 Réduit, Mauritius

## Abstract

Five traditionally used antidiabetic native medicinal plants of Mauritius, namely, *Stillingia lineata* (SL), *Faujasiopsis flexuosa* (FF), *Erythroxylum laurifolium* (EL), *Elaeodendron orientale* (EO), and *Antidesma madagascariensis* (AM), were studied for possible **α**-amylase and **α**-glucosidase inhibitory property, glucose entrapment, and amylolysis kinetics *in vitro*. Only methanolic extracts of EL, EO, and AM (7472.92 ± 5.99, 1745.58 ± 31.66, and 2222.96 ± 13.69 **μ**g/mL, resp.) were found to significantly (*P* < 0.05) inhibit **α**-amylase and were comparable to acarbose. EL, EO, AM, and SL extracts (5000 **μ**g/mL) were found to significantly (*P* < 0.05) inhibit **α**-glucosidase (between 87.41 ± 3.31 and 96.87 ± 1.37% inhibition). Enzyme kinetic studies showed an uncompetitive and mixed type of inhibition. Extracts showed significant (*P* < 0.05) glucose entrapment capacities (8 to 29% glucose diffusion retardation index (GDRI)), with SL being more active (29% GDRI) and showing concentration-dependent activity (29, 26, 21, 14, and 5%, resp.). Amylolysis kinetic studies showed that methanolic extracts were more potent inhibitors of **α**-amylase compared to aqueous extracts and possessed glucose entrapment properties. Our findings tend to provide justification for the hypoglycaemic action of these medicinal plants which has opened novel avenues for the development of new phytopharmaceuticals geared towards diabetes management.

## 1. Introduction

Phytomedicine also known as herbal medicine has become a mainstream phenomenon worldwide. Recently, it has been reported that more than 80% of the world population is dependent on herbal medicine [1]. The utilisation of plants and their derivatives for the treatment and/or management of various diseases, including diabetes mellitus (DM), is becoming more and more prominent in pharmaceutical markets as an alternative and/or complementary therapy. DM is a growing epidemic and is highly prevalent in Mauritius with at least one out of two adults aged between 25 and 74 years being prediabetic or diabetic [2, 3].

The fundamental defect in DM is the lack of insulin which results in the impairment in glucose uptake, storage, and utilisation [4]. Type 2 DM is the most common form of diabetes and is usually caused by life-style factors and also related to insufficient insulin production and resistance of target tissues to insulin. Several research works have been undertaken to elucidate the possible biochemical mechanisms involved in the pathogenesis of type 2 DM, but the exact mechanism is still unclear. However, hyperglycaemia, the hallmark of type 2 DM, has been considered as the principal cause of diabetes complications. Indeed, it was observed that strict glycaemic control lowered the incidence of retinopathy, nephropathy and neuropathy [5, 6].

Recently, there have been a growing number of scientific publications on the potential antidiabetic action of medicinal plants [7]. Indeed, advances in understanding the activity of key carbohydrate metabolising enzymes such as *α*-amylase and the role of dietary fibers have led to the development of new pharmacologic agents. Existing hypoglycemic agents such as metformin, voglibose, acarbose and miglitol effectively control glycemic level but carry prominent gastrointestinal side effects. The search for inhibitors devoid of side effects has been geared towards natural resources, namely, medicinal plants [8, 9]. Polyphenolic agents in plants have been shown to inhibit digestive enzymes due to their ability to bind to enzyme protein [10]. Moreover, the role of dietary fibres and viscous polysaccharides in the reduction of postprandial plasma glucose level in diabetic patients is highly documented [11].

The local population has a deep-rooted interest in the use of medicinal plants. Although a free advanced health care system exists, many Mauritians still rely on the use of folk medicine for the management of diabetes and related complications [7, 12]. Nonetheless, the majority of traditional antidiabetic medicinal plants await proper scientific and medical evaluation. In the present study selected medicinal plants of Mauritius were evaluated for their possible *α*-amylase and *α*-glucosidase inhibitory property, glucose movement entrapment and amylolysis kinetics effects using a battery of *in vitro* bioassays.

## 2. Materials and Method

### 2.1. Plant Materials and Extraction

Native traditionally used antidiabetic medicinal plants of Mauritius, namely, *Stillingia lineata* Lam. (Euphorbiaceae) (SL), *Faujasiopsis flexuosa* Lam. (Asteraceae) (FF), *Erythroxylum laurifolium* Lam. (Erythroxylaceae) (EL), *Elaeodendron orientale* Jacq. (Celastraceae) (EO), and *Antidesma madagascariensis* Lam. (Euphorbiaceae) (AM), were collected from a natural reserve situated on the upper humid regions of the island. The identity of the plants was confirmed by the natural reserve curator. The harvested plant materials were thoroughly washed under running tap water and air-dried until a constant weight was obtained. Subsequently, the dried samples were ground (Pacific mixer grinder, India) and stored in a cool-dry place prior to extraction. Crude methanolic extracts were obtained by soaking the dry powdered material into 70% methanol (1 : 10, sample : solvent w/v) for 72 h. Aqueous extracts, were prepared following traditional decoction method. Briefly, dried powdered material (50 g) was boiled into distilled water (200 mL) for 30 min. The filtrates were concentrated *in vacuo* using a rotary evaporator (Rotavap Stuart Scientific Ltd, Staffordshire, UK). The resulting paste-like material was stored at −20°C or dissolved in appropriate solvents.

### 2.2. *α*-Amylase Inhibition Assay

*α*-Amylase activity was assessed using the modified starch-iodine colour change method described previously by Mahomoodally et al. [9] and Kotowaroo et al. [13]. Briefly, 100 *μ*L *α*-amylase solution from porcine origin (13 U/mL in 0.1 M sodium acetate buffer pH 7.2) was added to 3 mL soluble starch solution (1 g soluble starch was suspended into 10 mL distilled water and boiled for 2 min. The volume was then made up to 100 mL with distilled water. The starch solution was used within 2-3 days) and 2 mL sodium acetate buffer (0.1 M, pH 7.2). The reaction mixture was incubated for 37°C for 1 h. At timed interval (*t* = 0 min and *t* = 60 min) aliquot (0.1 mL) from the reaction mixture was discharged into 10 mL iodine solution. After mixing, the absorbance of the starch-iodine solution was measured at 565 nm. As previously described [9] one unit of enzyme inhibitor was defined as that which reduced amylase activity by one unit and defined as [(*A*_0_ − *A*_*t*_)/*A*_0_] × 100; *A*_0_ and *A*_*t*_ being absorbance of starch-iodine solution at *t* = 0 min and *t* = 60 min, respectively. For assessing the potential inhibitory activity of graded concentrations of plant extracts (5000–312.5 *μ*g/mL) 100 *μ*L extract was preincubated with 100 *μ*L enzyme solution at 37°C for 15 min. The assay was then conducted as described above. Substrate and amylase blanks were carried out under similar assay conditions. The specific activity of amylase was described as U/mg protein/h.

### 2.3. Kinetics of *α*-Amylase Inhibition

A calibration curve using graded glucose concentration (10–0.156 mg/mL) was set up. Glucose solution (3 mL) was added to 3 mL dinitrosalicylic acid (DNS) reagent solution at 1% (10 g DNS, 0.5 g sodium disulphite, and 10 g sodium hydroxide) to capped tubes. The tubes were then placed in boiling water for 5–15 min until a reddish brown colour developed. Sodium potassium tartrate (1 mL, 40%) was then added to the mixture. After cooling, the absorbance was measured at 575 nm. The mode of inhibition of plant extracts on *α*-amylase action was determined by increasing the substrate (starch) concentration. The amount of glucose released after exactly 3 min was quantified using DNS reagent solution. 0.5 mL graded starch solution (4–0.25%), plant extract (0.25 mL; 5000 *μ*g/mL) and *α*-amylase solution (0.25 mL; 13 U/mL) were allowed to react for 3 min at 37°C. DNS solution (2 mL) was then added to stop the reaction and the mixture was placed in a boiling water bath for 5–15 min. Sodium potassium tartrate (1 mL, 40%) was then added and absorbance was measured at 575 nm using a spectrophotometer [13]. Kinetic parameters namely, the Michaelis-Menten constant affinity (*K*_*m*_) and maximum velocity (*V*_max⁡_), were derived from appropriate Lineweaver-Burk plots.

### 2.4. *α*-Glucosidase Inhibition Assay

*α*-Glucosidase inhibition was assessed using modified methods previously described by Bachhawat et al. [14] and Mayur et al. [5]. Briefly, 10 *μ*L *α*-glucosidase (1 U/mL), 50 *μ*L sodium phosphate buffer (0.1 M, pH 6.9), and 20 *μ*L *p*-nitrophenol-*α*-D-glucopyranoside (PNPG) substrate (1 mM) were incubated at 37°C for 30 min. After the incubation period, 50 *μ*L sodium carbonate (0.1 M) was added to the reaction mixture to terminate the reaction. The hydrolysis of PNPG to *p*-nitrophenol was monitored using an ELISA microplate reader at 405 nm. The IC_50_ value and % inhibition of glucosidase were calculated as % inhibition = [(Abs_blank_ − Abs_sample_)/Abs_blank_] × 100; Abs_blank_ is absorbance of the blank and Abs_sample_ is absorbance of the sample.

### 2.5. *α*-Glucosidase Kinetic Studies

The type of inhibition of plant extracts on *α*-glucosidase action was determined by increasing PNPG concentration following the modified method of Gurudeeban et al. [8]. Graded concentrations of *p*-nitrophenol (0.6–0.019 mM) were allowed to react with sodium carbonate and the absorbance was measured at 405 nm. Plant extract (20 *μ*L; 5000 *μ*g/mL) was incubated with 10 *μ*L *α*-glucosidase solution (1 U/mL), 50 *μ*L sodium phosphate buffer (0.1 M, pH 6.9), and 20 *μ*L graded concentrations of PNPG (1.25–0.039 mM) for 10 min at 37°C. The reaction was terminated by adding 50 *μ*L sodium carbonate (0.1 M). Kinetic parameters, namely, the Michaelis-Menten constants affinity (*K*_*m*_) and maximum velocity (*V*_max⁡_⁡), were derived from appropriate Lineweaver-Burk plots.

### 2.6. Glucose Movement

A simple model system was used to evaluate the effect of the plant extracts on glucose movement *in vitro*. This model was adapted from a method described by Shaukat et al. [15]. Briefly, the model used in the present experiment consisted of a one-sided sealed dialysis tube (15 cm × 25 mm, dialysis tubing membrane Sigma-Aldrich MW12173) into which 2 mL of 22 mM D-glucose in 0.15 M NaCl and 1 mL extract (160 mg/mL)/control (water) were incorporated. The other end was then sealed and the membrane was placed into a conical flask containing 45 mL 0.15 M NaCl. The conical flask was placed into an orbital shaking incubator (SI50, UK) at 37°C and speed of 100 rotations per minute. Aliquot (10 *μ*L) of the external solution was withdrawn at timed intervals and tested for the presence of glucose using a glucose oxidase kit (Biosystems, Spain). As described by Gallagher et al. [16] concentration-dependent effect of plant extracts (160, 80, 40, 20, and 10 mg crude extract/mL) that exhibited the highest glucose diffusion retardation index was also evaluated. A standard curve was drawn using different glucose concentrations. Experiments were conducted in triplicate. The glucose diffusion retardation index (GDRI) was calculated using the following formula.

GDRI = (100  −  glucose content (mg/mL) in external solution in the presence of plant extract/glucose content (mg/mL) in external solution in the absence of plant extract) ∗ 100.

### 2.7. Amylolysis Kinetics

This assay was adapted from Ahmed et al. [17]. Briefly, 8 g of soluble starch was dissolved in approximately 20 mL 0.1 M phosphate buffer (pH 6.5). The solution was boiled for 3 min and was made up to a final volume 100 mL to give an 8% (w/v) starch solution. The sample-*α*-amylase-starch system comprised extract (1 mL, 160 mg/mL), freshly prepared starch solution (3 mL, 8%), and enzyme solution (0.1% in 0.1 M phosphate buffer pH 6.5). The test system was dialysed against 45 mL distilled water at 37°C. The glucose concentration of the dialysate was monitored every hour for 4 h using a glucose oxidase kit (Biosystems, Spain). A control test was carried out with and without acarbose, a standard *α*-amylase inhibitor. After 4 h, the amount of starch remaining inside the dialysis tubing was quantified. To 5 mL iodine solution (0.254 g iodine and 4 g potassium iodide were dissolved in 1 L distilled water), 0.1 mL test mixture was added. The solution was vortexed and the absorbance was read at 565 nm. Then, using a calibration curve (4–0.125% starch solution) the amount of starch was quantified.

### 2.8. Statistical Analysis

Results were expressed as mean ± standard deviation of three independent determinations. Difference between the samples and controls was determined using one-way analysis of variance (ANOVA) with statistical significance considered as *P* < 0.05 using SPSS 16.0.

## 3. Results

### 3.1. *α*-Amylase Inhibition Assay

Data from the present study showed the variable inhibitory effect of tested plant extracts on *α*-amylase activity *in vitro*. Methanolic extracts of EL, EO, and AM were found to significantly (*P* < 0.05) inhibit *α*-amylase at different doses. IC_50_ values of extracts (methanolic EL, EO, and AM) are summarised in Table 1. As illustrated in Table 1, extracts activity (IC_50_ 1745.58–7472.92 *μ*g/mL) was found to be significantly lower compared to positive standard acarbose (1100 *μ*g/mL). In contrast, no dose-dependent response was observed for the other tested extracts (data not shown).

### 3.2. *α*-Amylase Kinetic Studies

Since activity was observed for EL, EO, and AM methanolic extracts, kinetic studies were performed on these extracts. Methanolic EO and AM extracts showed an uncompetitive type of inhibition, whereby there was a reduction in both *K*_*m*_ and *V*_max⁡_⁡. As presented in Table 2, in the presence of EO *K*_*m*_ was reduced from 3.73 × 10^−1^ mg to 3.05 × 10^−1^ mg and *V*_max⁡_⁡ from 0.03 × 10^−1^ mg mL^−1^ sec^−1^ to 0.01 × 10^−1^ mg mL^−1^ sec^−1^. Similarly, *K*_*m*_ was reduced from 4.98 × 10^−1^ mg to 3.63 × 10^−1^ mg and *V*_max⁡_⁡ from 0.04 × 10^−1^ mg mL^−1^ sec^−1^ to 0.03 × 10^−1^ mg mL^−1^ sec^−1^ in the presence of methanolic AM. In contrast, in the presence of EL, *K*_*m*_ was raised from 3.73 × 10^−1^ mg to 4.37 × 10^−1^ mg while *V*_max⁡_⁡ was reduced to 0.02 × 10^−1^ mg mL^−1^ sec^−1^.

### 3.3. *α*-Glucosidase Inhibition *In Vitro*

*α*-Glucosidase activity was assessed by the release of *p*-nitrophenol from PNPG *in vitro*. IC_50_ (*μ*g/mL) values of active extracts are presented in Table 3. Tested extracts exhibited various levels of effectiveness in inhibiting *α*-glucosidase. It was observed that both methanolic and aqueous extracts of EL, EO, AM, and SL were potent inhibitors (1.02–185.92 *μ*g/mL) of *α*-glucosidase compared to acarbose (5115.73 *μ*g/mL).

### 3.4. *α*-Glucosidase Kinetic Studies

Table 4 presents the *V*_max⁡_⁡ and *K*_*m*_ values of active plants extracts against *α*-glucosidase. A decrease in both *K*_*m*_ and *V*_max⁡_⁡ as compared to the uninhibited reaction (61.40 × 10^−2^ mM (*K*_*m*_), 2.50 × 10^−2^ mg mL^−1^ sec^−1^ (*V*_max⁡_)) was noted for all tested extracts.

### 3.5. Glucose Movement

**Figure 1:**
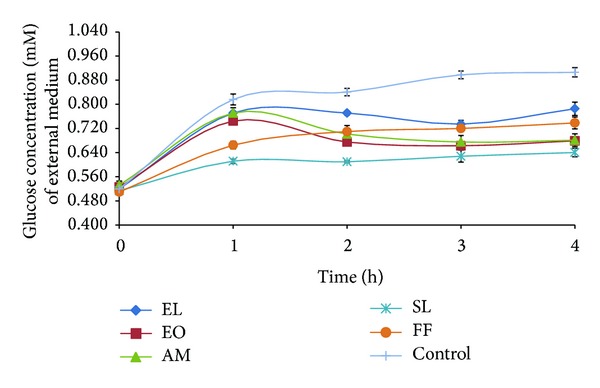
Effect of methanolic plant extracts (160 mg crude extract/mL) on glucose diffusion.

**Figure 2:**
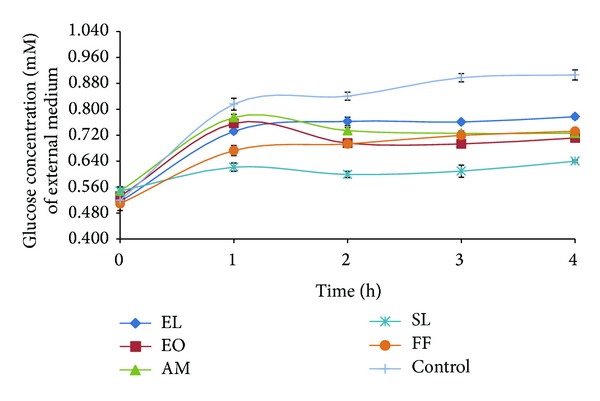
Effect of aqueous plant extracts (160 mg crude extract/mL) on glucose diffusion.

Glucose movement for the control experiment (without plant extract) showed a mean glucose concentration of 0.906 mM. From Figures 1 and 2, it was observed that there was no apparent difference in glucose diffusion inhibition between the different types of extracts. As shown in Table 5, studied extracts exhibited glucose diffusion retardation index (GDRI) between 8 and 29%. Furthermore, it was observed that methanolic extracts were more potent inhibitors of glucose movement.

**Figure 3:**
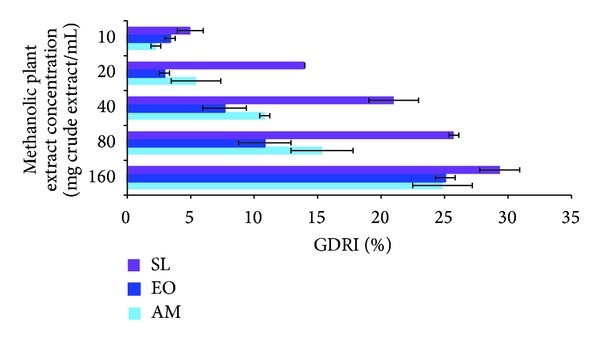
Dose-dependent effect of SL, EO, and AM extracts on glucose diffusion.

Dose-dependent studies on the effect of extracts on glucose retarding activity revealed a concentration-dependent inhibitory action (Figure 3). GDRI (%) decreased with decreasing plant extract concentration. SL was found to exhibit greater GDRI at all concentrations tested.

### 3.6. Amylolysis Kinetics

**Figure 4:**
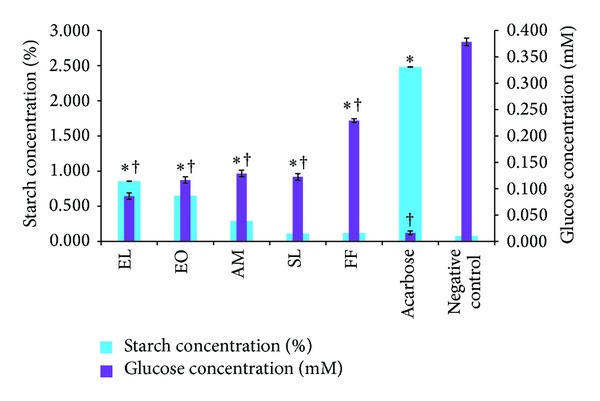
Percentage starch of reaction mixture and glucose concentration of dialysate in the presence of aqueous extracts. *Values [starch (%) concentration] significantly (*P* < 0.05) higher than negative control. ^†^Values [glucose (mM) concentration] significantly (*P* < 0.05) lower than negative control.

**Figure 5:**
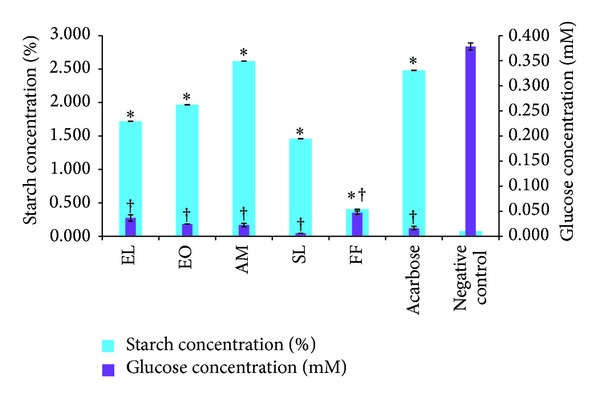
Percentage starch of reaction mixture and glucose concentration of dialysate in the presence of methanolic extracts. *Values [starch (%) concentration] significantly (*P* < 0.05) higher than negative control. ^†^Values [glucose (mM) concentration] significantly (*P* < 0.05) lower than negative control.

Figures 4 and 5 summarise the starch concentration (%) of the reaction mixture inside the dialysis bag and the glucose concentration (mM) of the surrounding solution after 4 h. Methanolic extracts were found to be potent inhibitors compared to their corresponding aqueous extracts. As observed by the *α*-amylase inhibition assay, methanolic EL, EO, and AM gave the best inhibitory activity since starch concentration was the highest in the presence of these extracts (Figure 5). Glucose dialysis was the least in the presence of methanolic SL extract.

## 4. Discussion

The present study was geared towards investigating the potential effects of selected medicinal plants of Mauritius to inhibit key carbohydrate hydrolysing enzymes, namely, *α*-amylase and *α*-glucosidase. Furthermore, the ability of the extracts to entrap glucose and amylolysis kinetics were also evaluated. *α*-Amylase and *α*-glucosidase are key carbohydrate hydrolysing enzymes responsible for breaking *α*,1-4 bonds in disaccharides and polysaccharides, liberating glucose [18, 19]. The glucose surge observed a few minutes after ingestion contributes to hyperglycaemia, the hallmark of DM. Several scientific studies have shed light on the inhibition of these key glycoside hydrolases to slow down carbohydrate digestion, reducing glucose absorption rate, consequently preventing postprandial glucose surge [20, 21]. The ability of plant extracts to modulate glucose liberation from starch and its absorption [10] has proved to be an attractive therapeutic modality in the management of DM. Polyphenolic compounds found in extracts have also been reported to interact with proteins and hence inhibit enzymatic activity [10, 22].

Results from this study tend to show that extracts of selected medicinal plants showed variable inhibitory effect on *α*-amylase and *α*-glucosidase *in vitro*. It was observed that three methanolic extracts (EL, EO, and AM) possessed dose-dependent *α*-amylase inhibitory activity. From data amassed, it was obvious that methanolic fractions carried higher concentration of inhibitory phytochemicals as previously reported [9, 23]. Furthermore, several scientific reports highlight the inhibitory action of plant phytochemicals on *α*-amylase [23, 24]. Additionally, the kinetic model of these extracts on *α*-amylase was studied and it was found that in the presence of methanolic extracts of EO and AM, a decrease in both *K*_*m*_ (the affinity of the enzymes for the substrate) and *V*_max⁡_⁡ (the velocity of reaction) were observed. This tends to suggest an uncompetitive mode of inhibition. Uncompetitive inhibitors bind to enzyme-substrate complex forming an enzyme-substrate-inhibitor complex [25, 26]. This complex reduces affinity for the enzyme active site for the substrate decreasing the affinity and delays rate of reaction [14, 27]. It was also noted that active extracts uncompetitively inhibited *α*-glucosidase. Furthermore, *α*-glucosidase inhibitory assay tends to show that extracts of medicinal plants were potent inhibitors of *α*-glucosidase as compared to acarbose. This finding was consistent with Shai et al. [28] who reported the little inhibitory action of acarbose on *α*-glucosidase. In contrast, methanolic extract of EL was found to follow mixed type of inhibition. Mixed inhibitor bind to free and to substrate bound enzyme and interfere with binding and catalysis of substrate [25, 26], increasing affinity and decreasing reaction rate [27]. Retarding glucose production and/or absorption might be important strategies in the management of diabetes.

We also investigated the effect of selected medicinal plants on glucose entrapment *in vitro*. A number of studies have unravelled the value of plants complex polysaccharides such as guar gum, oats, and psyllium husk in lowering blood glucose level [28]. The retardation in glucose diffusion *in vivo* might be attributed to the physical obstacles, insoluble fibre particles, which entrap glucose molecules within the fibre network preventing postprandial glucose rise [17, 29]. They form a viscous matrix which delay gastric emptying and slow glucose uptake [17, 30]. The viscous gel also impedes the access of glucose to the small intestines' epithelium, blunting postprandial glucose peaks. GDRI, a useful *in vitro* index to predict the effect of fibres present in the extracts on the delay in glucose absorption, was calculated in this study [24, 31]. SL was found to have the highest GDRI value. Similarly, Wood et al. [32] reported that plants showing between 6 and 48% inhibitory action on glucose diffusion across a semipermeable membrane possessed moderate inhibitory activity. Furthermore, widely studied sources of soluble fibres such as wheat bran, oats, and psyllium husk were found to inhibit between 10 and 23% glucose diffusion after 180 min *in vitro* [31]. However, in the present study we observed that SL was a poor *α*-amylase inhibitor. It could be argued that the antidiabetic action of SL might be due to this glucose movement retardation properties rather than *α*-amylase inhibition. Further studies demonstrated that glucose movement retardation properties were dose dependent. Published literature highlight the effect of soluble fibre's molecular weight and concentration along with viscosity on modulating glucose dialysis [11, 33]. Another possible mechanism is the sequestration of enzymatic activity on carbohydrates. As reported from previous study amylolysis assay showed that the retardation of glucose diffusion is also due to the inhibition of *α*-amylase, thus limiting the release of glucose from starch [31]. The inhibition of *α*-amylase might be due to the concerted action of encapsulation of the enzyme and/or starch in the fiber matrix and/or the action of inhibitors. Eventually, this leads to reduced glucose absorption and blunting of postprandial glucose rise [27].

## 5. Conclusion

The present study demonstrated the ability of native antidiabetic medicinal plants of Mauritius to inhibit key carbohydrate hydrolysing enzymes and unravelled their mode of inhibition. Furthermore, to date no such study has been conducted to evaluate the glucose entrapment properties and amylolysis kinetic effects of these extracts. Data gathered suggest that methanolic fractions of EO, EL, and AM were active enzyme inhibitors. Pertaining to the role of these enzymes in the control of post-prandial increase of blood glucose level, their inhibition could be useful in the development of new drug strategies. Further scientific validation is essential to understand the therapeutic potential of these medicinal plants for improving glycaemic control in diabetic subjects and confirm their antidiabetic mode of action.

## Figures and Tables

**Table 1:** IC_50_ values of methanolic plants against *α*-amylase.

Plant extracts	IC_50_ value (µg/mL)
EL	7472.92 ± 5.99^a^
EO	1745.58 ± 31.66^a^
AM	2222.96 ± 13.69^a^
Control	1100.06 ± 0.03

Data represents the mean ± standard deviation of triplicate values. ^a^Values significantly lower (*P* < 0.05) than positive control (acarbose).

**Table 2:** Kinetic parameters of active plant extracts on **α**-amylase activity *in vitro. *

Plants extracts (5000 µg/mL)	*K*_*m*_ (mg ×10^−1^)	*V*_max⁡_ (mgmL^−1^sec^−1^ ×10^−1^)
EL	4.37	0.02
EO	3.05	0.01
AM	3.63	0.03

**Table 3:** IC_50_ values (µg/mL) of methanolic and aqueous plants extracts that actively inhibit **α**-glucosidase.

Plant extracts	IC_50_ value (µg/mL)
EL	[1.02 ± 0.02^b^]
	(12.00 ± 1.57^b^)
EO	[1.75 ± 0.26^b^]
	(16.72 ± 2.81^b^)
AM	[10.40 ± 0.26^b^]
	(1.22 ± 0.05^b^)
SL	[19.30 ± 3.59^b^]
	(185.92 ± 9.00^b^)
Control	5115.73 ± 3.91

^b^Values significantly (*P* < 0.05) lower than control (acarbose); [] methanolic extracts; ( ) aqueous extracts.

**Table 4:** Kinetic parameters of methanolic and aqueous plant extracts on **α**-glucosidase activity *in vitro. *

Plant extracts (5000 µg/mL)	*K*_*m*_ (mM ×10^−2^)	*V*_max⁡_ (mMmin^−1^ ×10^−2^)
EL	[0.60]	[0.90]
	(0.50)	(1.20)
EO	[0.70]	[0.70]
	(0.80)	(0.70)
AM	[0.80]	[0.90]
	(2.40)	(0.50)
SL	[2.60]	[0.50]
	(1.00)	(2.00)

[] methanolic extracts; ( ) aqueous extracts.

**Table 5:** Glucose concentration in external solution and glucose diffusion retardation index of methanolic plant extracts after 4 h.

Plant extracts	Glucose concentration in external solution^1^ (mM)	GDRI^2^ (%)
EL	[0.785 ± 0.022^c^]	[13 ± 2.44]
	(0.777 ± 0.007^c^)	(14 ± 0.78)
EO	[0.679 ± 0.007^c^]	[25 ± 0.78]
	(0.712 ± 0.011^c^)	(21 ± 1.17)
AM	[0.681 ± 0.021^c^]	[25 ± 2.35]
	(0.726 ± 0.007^c^)	(20 ± 0.78)
SL	[0.640 ± 0.014^c^]	[29 ± 1.56]
	(0.640 ± 0.004^c^)	(29 ± 0.39)
FF	[0.738 ± 0.020^c^]	[19 ± 2.18]
	(0.732 ± 0.009^c^)	(19 ± 0.78)
Control	0.906 ± 0.015	—

^1^Values are mean ± SD of triplicate determinations; ^c^values significantly (*P* < 0.05) different from negative control; ^2^GDRI expressed as percentage; GDRI ± SD was calculated from triplicate determinations; [] methanolic extracts; ( ) aqueous extracts.
